# Introducing a revised version of the Kumamoto scale as an easy-to-use clinical tool for monitoring multisystemic changes in hereditary transthyretin amyloidosis

**DOI:** 10.1186/s13023-025-03915-w

**Published:** 2025-07-25

**Authors:** Jonas Wixner, Björn Pilebro, Tale N. Wien, Per Eldhagen, Henning Mölgaard, Björn Hedström, Astrid J. Terkelsen

**Affiliations:** 1 Department of Public Health and Clinical Medicine, University Hospital of Umeå, Umeå University, 901 85 Umeå, Sweden; 2 Department of Internal Medicine, Baerum Hospital, Vestre Viken HF, Drammen, Norway; 3 Department of Medicine, Karolinska Institute, Stockholm, Sweden; 4 Department of Cardiology, Aarhus University, Aarhus, Denmark; 5 Department of Internal Medicine, Ängelholm Hospital, Ängelholm, Sweden; 6 Department of Neurology, Aarhus University Hospital and Danish Pain Research Center, Clinical Medicine, Aarhus University, Aarhus, Denmark

**Keywords:** Hereditary amyloidosis, Transthyretin, Registry, Liver transplantation, Drug therapies

## Abstract

**Background:**

Hereditary transthyretin (ATTRv) amyloidosis is a rare but life-threatening multisystemic disease. Multiple disease-modifying treatments are now available and standardised instruments for early detection and disease monitoring are essential. Still, validated and easy-to-use tools for clinical follow-up are scarce.

**Methods:**

The Kumamoto scale was first described in 1997 as a method for systematically evaluating patients with ATTRv amyloidosis and has been used in clinical trials since. A panel of amyloidosis experts from Sweden, Denmark, and Norway discussed the strengths and limitations of the Kumamoto scale at the Nordic Amyloidosis Day at Arlanda in 2023, and it was decided to revise and improve the scale that has been used in routine clinical monitoring of patients in Sweden since 2020. Our aim is to introduce the revised version of the Kumamoto scale as a useful clinical monitoring tool.

**Results:**

Minor adjustments were applied to make the scale more sensitive and precise. Bedside instruments for sensory examination were defined as well as the sensory and motor levels. Constipation was added as a sign of autonomic dysfunction. The subtotal and total scores remain unchanged.

**Conclusions:**

We believe that the revised Kumamoto scale is a reliable and easy-to-use clinical tool for monitoring ATTRv amyloidosis.

## Introduction

Hereditary transthyretin (ATTRv) amyloidosis is a rare but life-threatening multisystemic genetic disease associated with transthyretin (TTR) gene variants, of which the V30M (*p.V50*) and the V122I (*p.V142I*) variants are the most frequent [1]. Common disease complications include autonomic and sensorimotor polyneuropathy, neuropathic pain, gastrointestinal disturbances, cardiac arrhythmias, cardiomyopathy, visual disturbances, and renal failure. Both the genotype and the amyloid fibril type have been linked to the phenotype and to the prognosis of the disease [2]. Natural history data have shown that the median survival time without treatment ranges from 7 to 12 years after disease onset [3–7], with a reduced survival time for patients presenting with cardiomyopathy [3, 8, 9]. Thanks to breakthroughs in the field, several disease-modifying therapies have emerged during the last decades. Liver transplantation was introduced in 1990 and has since been a successful treatment for many patients [10]. More recently, disease-modifying drugs have been introduced, and both TTR stabilizers [11–15] and TTR gene silencers [16–20] have been shown to reduce or halt disease progression. However, not all patients have the same beneficial effect from these treatments [10–12, 17], and many of the available drugs are very expensive [21, 22]. Thus, standardized and efficient instruments for disease monitoring are essential but, still, validated and easy-to-use tools for clinical follow-up remains scarce.

For patients with ATTR cardiomyopathy, overall survival and cardiac-related hospitalisations as well as 6-min-walk tests, cardiac biomarkers, and echocardiographic findings have been used for treatment evaluation in clinical trials [14, 15, 20, 23]. Moreover, a combination of nt-proBNP and serum creatinine can be used as a simple prognostic tool in the same population [24]. In clinical trials for polyneuropathy patients, different versions of the Neuropathy Impairment Score (NIS), NIS + 7 and the modified NIS + 7 (mNIS + 7) have been used to evaluate disease progression [11, 13, 16–19]. NIS, or at least NIS lower limb (NIS-LL), is fairly easy to use in clinical practice but its use is limited to the evaluation of peripheral large fibre polyneuropathy [25]. NIS + 7 and mNIS + 7 are also used to evaluate autonomic neuropathy to some extent [25], but may be too complicated or time-consuming for use in clinical practice. There are some recently published national and international guidelines with recommendations on the management and follow-up of patients with ATTRv amyloidosis; however, so far, there is no firm consensus on which scores and scales that should be used [26–31].

The Kumamoto scale was first described in 1997 as a method for systematically evaluating patients with ATTRv amyloidosis [32] and has, together with other outcome measures, been used in clinical trials since then [13, 33]. It has also been used for evaluating the outcome after liver transplantation in patients with ATTRV30M amyloidosis and polyneuropathy [34]. The Kumamoto scale covers the sensory, motor, and autonomic function of patients as well as visceral organ involvement, and the instrument is based on a clinical examination together with ECG and laboratory findings. Like every scale it is not perfect, but it covers most disease complications and is easy to use in clinical practice. In Sweden, the Kumamoto scale has been used for routine clinical follow-up of ATTRv patients since 2020, and the scale is recommended in the national Swedish treatment guidelines [35]. In Denmark, the scale is recommended by the Danish Medicines Council to follow disease progression in patients with ATTRv polyneuropathy during disease-modifying treatment [36]. Having used the scale at the clinic in recent years, we have noticed that some elements of the scale could benefit from slight adjustments. We therefore introduce a revised version of the Kumamoto scale as a useful clinical monitoring tool for ATTRv amyloidosis. We present and discuss the original Kumamoto scale in detail with focus on its strengths and limitations as well as suggest improvements and present a revised version.

## Methods

### Strengths and limitations of the original Kumamoto Scale

**Fig. 1:**
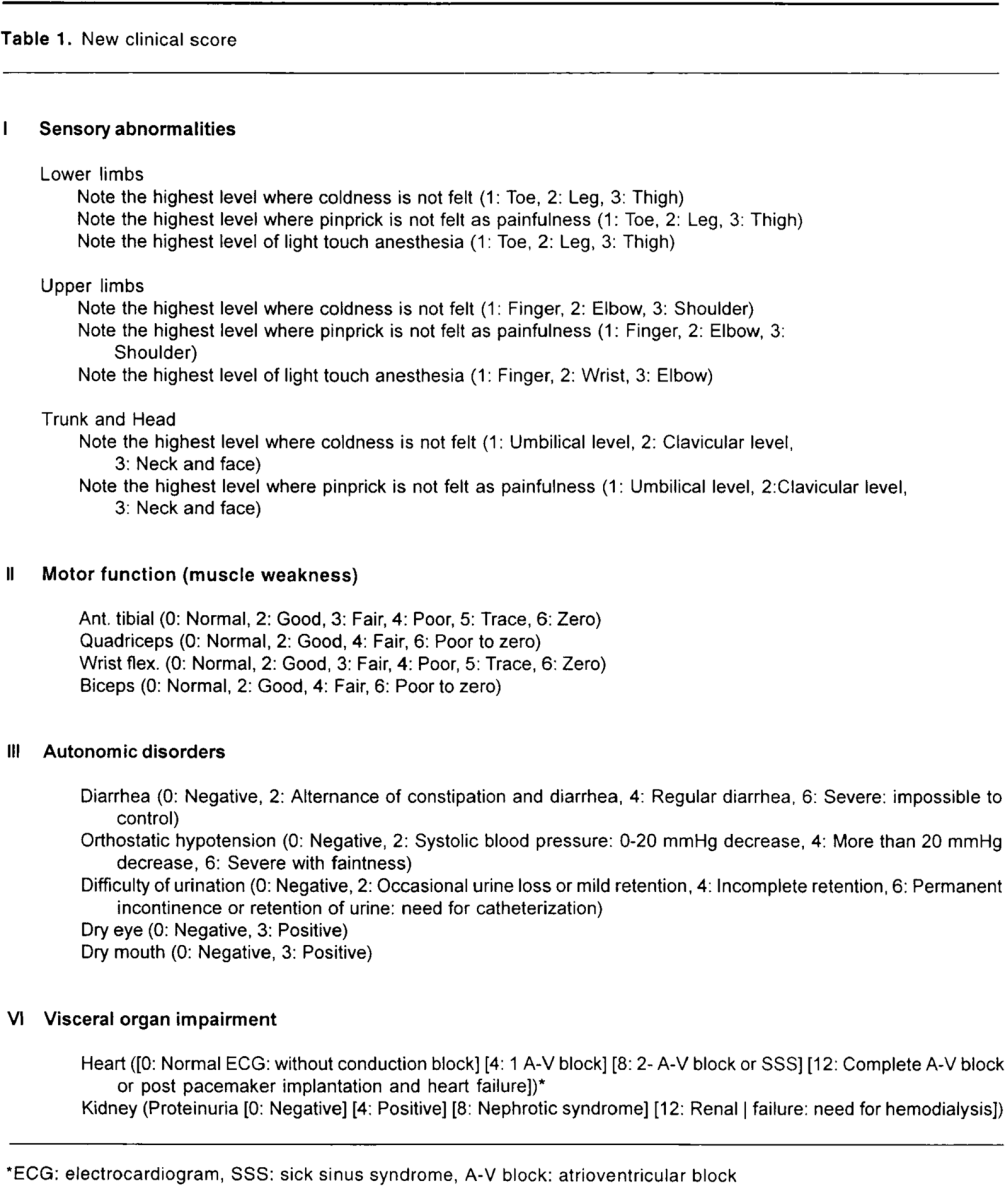
The original version of the Kumamoto scale, published in Amyloid in 1997 [32]

The original Kumamoto Scale [32] is presented in Fig. 1. It consists of four subdomains, including sensory abnormalities, motor function, autonomic disorders, and visceral organ impairment, which all have a score ranging from 0 to 24 points, summing up to a total score ranging from 0 to 96 points. Below, we discuss the strengths and limitations of the original scale based on discussions by a panel of amyloidosis experts with different medical specialities from Sweden, Denmark, and Norway at the Nordic Amyloidosis Day at Arlanda, Sweden in 2023. At the meeting, a decision was made to revise and improve the Kumamoto scale.

#### Scoring of sensory abnormalities

Sensation to cold, pinprick, and light touch is assessed in the Kumamoto scale, for which bedside test batteries have been evaluated in a number of studies [37–40]. Cutaneous sensory neurons are classified as either Aβ, Aδ, or C fibres. Slow and persistent pain is transmitted by unmyelinated C fibres that are the smallest nerve fibres with the slowest conduction velocities. Immediate pain is transmitted by thinly myelinated Aδ fibres with intermediate conduction velocity. Aβ sensory neurons are heavily myelinated, thereby exhibiting rapid conduction velocity. Most Aβ fibres have low mechanical thresholds, performing as light-touch receptors.

Cold sensation can be tested by asking the patients if they can feel the coldness when applying a cold tuning fork or a metal coin on the skin or by using the TipTherm (Gesellschaft für neurologische Diagnostik, Düsseldorf, Germany), which is a small pocket-sized device, feasible for quick punctual thermal sensory testing. A metal area serves as a cold and a plastic area as a warm stimulus [41]. Rolltemp II (Somedic SenseLab AB, Sweden) is designed for quick screening of temperature sensibility over large body areas with a predetermined temperature level of 25 °C for cold and 40 °C for warmth. The skin temperature should be 32 °C.

Pinprick can be tested with a broken cotton bud or a needle. Standardised testing for pinprick with Neuropen® with NeuroTip (Owen Mumford, Oxford, UK) using sterile, single-use neurological examination pins eliminates the risk of cross infection and skin puncture [42]. A calibrated force of 40 g may be safely exerted onto the skin to identify patients with decreased sensation to sharpness and pain in small nerve fibres. Finally, light touch can be tested with cotton.

Sensory amyloid-related neuropathy may be length dependent with a glove-and-sock distribution. However, in some cases it has a patchy or widespread manifestation [43]. Consequently, assessment scales such as the Utah Early Neuropathy Scale [44] designed for length dependent neuropathy will not detect the patchy or generalised presentation. The key feature and strength of the Kumamoto scale is its systematic and quantitative evaluation of whole-body sensory disturbances that also can detect atypical presentations. Nonetheless, we suggest some adjustments since the tools for sensory examination has not been closely defined and since the anatomical levels for sensory testing are somewhat imprecise (i.e., toe, leg, and thigh).

Although the sensory modalities are transmitted by different types of nerve fibres, one feature of the Kumamoto scale that may be confusing is that the areas used for rating sensory abnormalities in the upper limb differ between cold sensation/pinprick and light touch.

Another ambiguity is that the scale aims to follow the progression of neuropathy but, for the limbs, the most proximal area of lost sensation is marked as “leg”, “thigh”, “elbow”, and “shoulder”, which is confusing because the thigh is the upper part of the leg, and the proximal borders of the elbow and the shoulder are not precisely defined. Also, the highest level of lost sensation on the trunk and head mentioning the “umbilical level”, “clavicular level”, and “neck and face” must be questioned as such abnormalities relate to medullary dermatomal changes. Truncal polyneuropathy is a clinical entity characterised by sensory deficit in the distribution of the thoracic intercostal nerves. The sensory loss is relatively symmetric and involves multiple thoracic dermatomes, beginning close to the anterior midline. This entity is important because it can be confused with myelopathies that produce sensory levels over the torso [45]. Thus, peripheral neuropathy will affect the end of the intercostal nerves and the midline of the trunk and spreading toward the periphery rather than distal to proximal from the umbilical area to the clavicular level and face.

Finally, it is difficult to differ between reduced and normal sensation, while a loss of sensation is clearer for both the patient and the examiner. There is a discrepancy between the two publications defining the Kumamoto scale from respectively 1997 [32] and 1999 [34], as the first paper reports a loss of sensation, whereas the second reports reduced sensation. We recommend that *loss of sensation* is used.

#### Scoring of autonomic disorders

##### Diarrhoea

Constipation is usually the first lower GI symptom of the disease [46]; however, it is not a separate symptom in the original Kumamoto scale. Further, no upper GI symptoms are included. It is important to note that diarrhoea is generally defined as three or more loose stools per day, which has not been clearly stated.

##### Orthostatic hypotension

According to guidelines for measuring autonomic function [47], a reduction in systolic and diastolic blood pressure of 20 and 10 mm Hg, respectively, defines orthostatic hypotension unless the patient has supine hypertension. In that case, the systolic blood pressure reduction should be at least 30 mm Hg. Normally, orthostatic hypotension should be evaluated within 3 min of standing [47]. The original version of the Kumamoto scale includes a systolic blood pressure reduction of 0–20 mm Hg. It can be discussed whether such small reductions are of any clinical relevance unless the patient has previously experienced increased blood pressure while standing up. Further, a description on how orthostasis should be evaluated has not been provided.

##### Difficulty of urination

As regards mild and incomplete urinary retention, it is not mentioned that double voiding followed by a bladder scan should be performed. Patients are not always able to feel a mild or incomplete urinary retention. Moreover, definitions of mild and incomplete retention are lacking. It is also worth mentioning that benign prostate hyperplasia should be excluded as a cause of urinary retention in elderly males.

##### Dry eye and mouth

Dry mouth, which together with dry eyes is part of the Sicca syndrome, is a common autonomic complication of ATTRv amyloidosis, but it is also a common side effect of many drugs used for symptomatic treatment of the disease. It is therefore important to exclude that the dry mouth is a side effect of the patient’s medications.

#### Scoring of motor functions (muscle weakness)

The following muscles are listed in the original scale: tibialis anterior, quadriceps, and biceps; however, also movements are listed in this part of the scale (wrist flexion). Further, it is not feasible to make one motor score for the quadriceps femoris muscle as it both extends the knee and flexes the hip. Muscle weakness needs to be tested on single and not multiple joints. Scoring of muscle power also needs to follow the suggested international guidelines, i.e., NIS (0–4) [25] or the Medical Research Council scale (MRC, 0–5) [48].

#### Scoring of visceral organ impairment

Cardiac involvement focuses on conduction disturbances, although cardiomyopathy and heart failure are also quite common in ATTRv amyloidosis. Scoring for heart failure is only included if the patient also has a third-degree atrioventricular (AV) block or a pacemaker. Precise definitions of nephrotic syndrome and renal failure are lacking.

## Results

### Revision of the Kumamoto scale

#### Scoring of sensory abnormalities

For use in multicentre studies, the tools used for the sensory examinations need to be defined, which is not specified in the original version of the Kumamoto scale. Sensory examinations are not easy to perform since they are partly subjective and therefore need to be standardised.

##### Precise description of bedside instruments for examination

To improve the reliability of the evaluation, we suggest to rate loss of sensation to cold, pinprick, and light touch instead of reduced sensation in accordance with the first publication on the Kumamoto scale [32]. Cold detection should be examined with the Rolltemp II (Somedic SenseLab AB, Sweden) with predetermined temperature levels at 25 °C. The limb needs to be heated if colder than 25 °C. Pinprick should be tested with a Neuropen® with NeuroTip (Owen Mumford, Oxford, UK). Light touch should be tested with cotton balls.

##### Definition of areas for sensory dysfunction

**Fig. 2:**
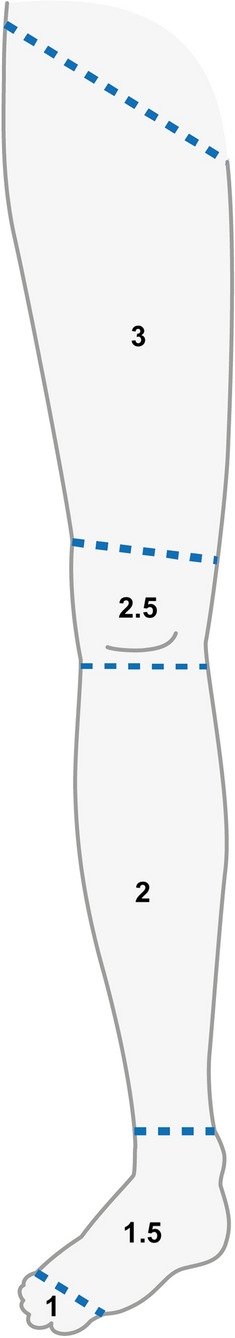
Lower limb sensation. Change from “toe”, “leg”, and “thigh” to “toe”, “foot”, “lower leg”, “knee”, and “thigh”. Same areas for all modalities: Cold, pinprick, and light touch. Suggested new scoring for loss of sensation: 0 = normal sensation, 1 = one or all toes, 1.5 = foot (base of toes to top of malleolus), 2 = lower leg (top of malleolus to bottom of kneecap), 2.5 = knee (bottom to top of kneecap), 3 = thigh (top of kneecap to groin)

**Fig. 3:**
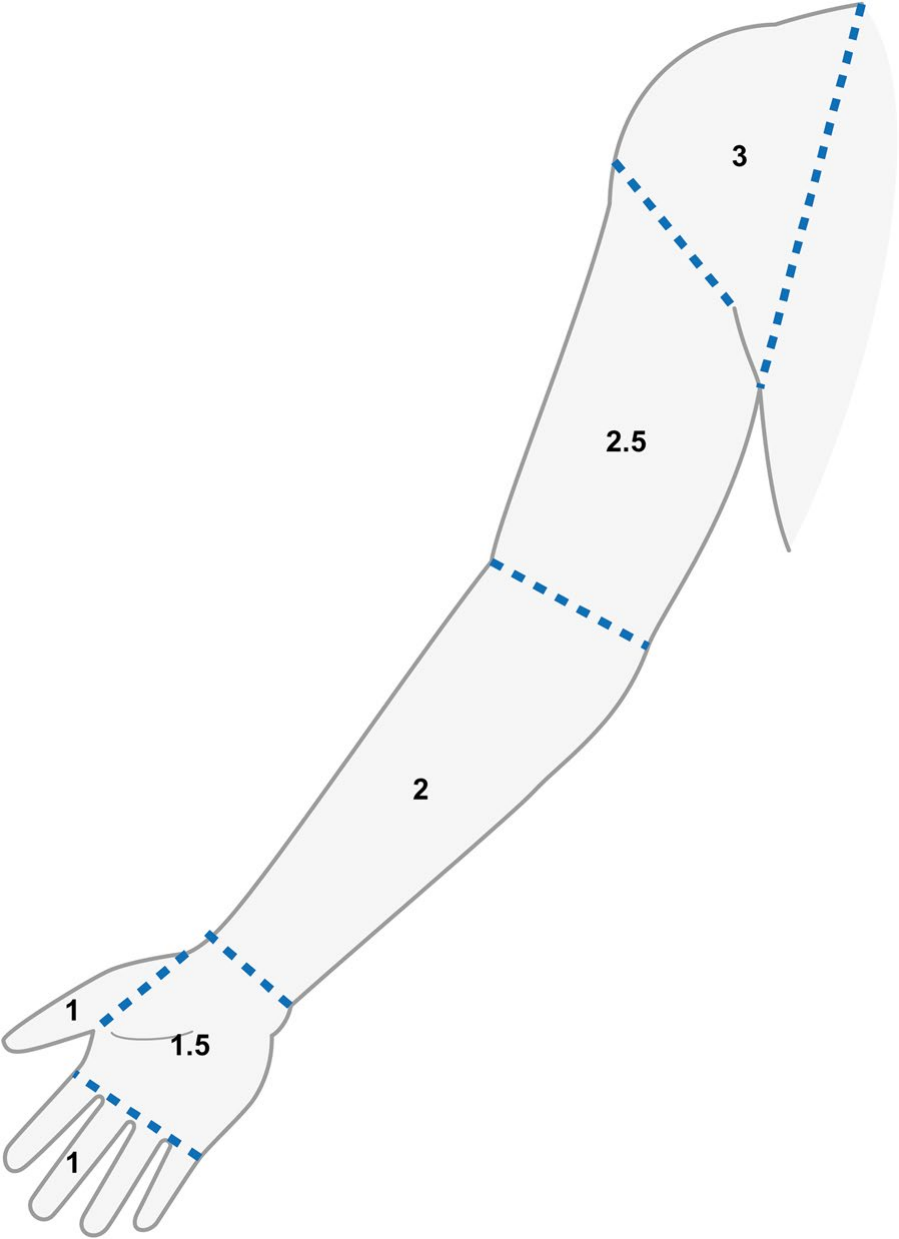
Upper limb sensation. Change from “finger”, “elbow”, and “shoulder” to “finger”, “hand”, “forearm”, “upper arm”, and “shoulder”*.* Same areas for all modalities: Cold, pinprick, and light touch. Suggested new scoring for loss of sensation: 0 = normal sensation, 1 = one or all fingers, 1.5 = hand (base of fingers to processus styloideus), 2 = forearm (processus styloideus to elbow bending), 2.5 = upper arm (elbow bending to acromion), 3 = shoulder (acromion to axillar line)

**Fig. 4:**
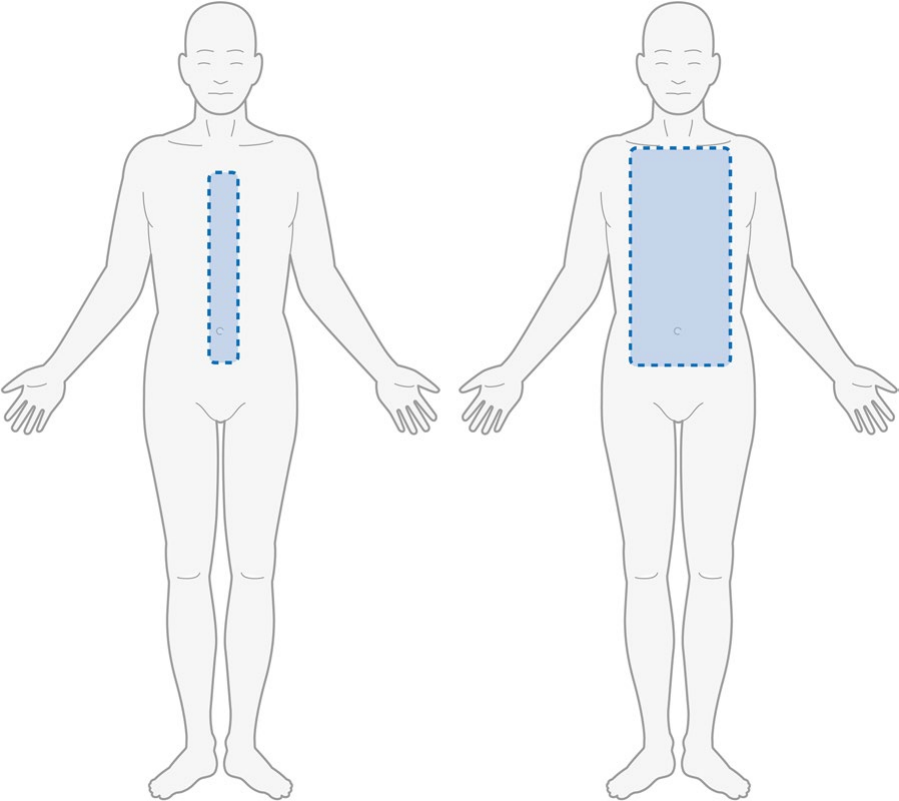
Trunk and head sensation. Change from “umbilical level”, “clavicular level”, and “neck and face” to “midline trunk”, "midclavicular line", and "neck and face”. Scoring for loss of sensation remains unchanged (1, 2, and 3 for each level, respectively)

In Figs. 2, 3, and 4, the areas and scoring for sensory dysfunction are redefined to make the scale more granular. Instead of the original three levels for sensory evaluation in the upper and lower extremities, the revised version contains five sensory levels, which have also been more closely defined in relation to anatomical structures (Figs. 2, 3). For each new sensory level (foot, knee, hand and upper arm), 0.5 point was added to the score. Thus, the score for each sensory domain (cold, pin prick and light touch) still ranges from 0 to 3 points, but with six different sub-scores (0, 1, 1.5, 2, 2.5 and 3) instead of four (0, 1, 2 and 3), which makes it easier to capture a more subtle progression of sensory neuropathy.

The sensory evaluation of the trunk and head has been redefined (Fig. 4) to reflect the distribution of intercostal neuropathy starting distally (from the midline) and spreading proximally (to the periphery), but the scoring was left unchanged (0, 1, 2 or 3 points).

#### Scoring of autonomic disorders

##### Diarrhoea

We suggest changing the item name from “Diarrhoea” to “GI symptoms” and adding constipation as a separate symptom (1 point) to reflect early lower GI dysfunction. Constipation is generally defined as fewer than three (hard) stools per week. Upper GI symptoms should also be considered, but they are difficult to add to the scale without interfering too much with the original scoring. Thus, the scoring still ranges from 0 to 6 points but with five subscores (0, 1, 2, 4 and 6) instead of four (0, 2, 4 and 6).

##### Orthostatic hypotension

We suggest changing the 2-point systolic blood pressure interval from 0–20 mm Hg to 10–20 mm Hg to detect early signs of cardiovascular adrenergic dysfunction. Orthostatic hypotension should be evaluated with blood pressure measurements after 5 min of rest in a supine position, and then after 1 and 3 min in a standing position [47]. Other causes of hypotension (antihypertensives, etc.) should be considered when interpreting these results. The scoring was left unchanged ranging from 0 to 6 points with four subscores (0, 2, 4 and 6).

##### Difficulty of urination

We suggest changing the item from “Difficulty of urination” to “Urination problems”. The different stages of urinary retention have been defined according to the amount of residual urine and in accordance with current Swedish clinical practice [49]. Double voiding followed by a bladder scan should be performed in all cases. The scoring was left unchanged ranging from 0 to 6 points with four subscores (0, 2, 4 and 6), where less than 150 ml of residual urine equals 0 points, sporadic incontinence or 150–300 ml of residual urine equals 2 points, urinary retention with 300–500 ml of residual urine equals 4 points, and catheterization or >500 ml of residual urine equals 6 points.

#### Scoring of motor functions (muscle weakness)

Tibialis anterior muscle, quadriceps muscle, biceps muscle, and wrist flexion are changed to ankle dorsiflexion, knee extension, elbow flexion, and wrist dorsiflexion. International standards should be used, preferably the Medical Research Council (MRC) scale with some modifications according to the Kumamoto scale (Table 1). Apart from applying the MRC scale for evaluation, the scoring was left unchanged ranging from 0 to 6 points with six sub-scores (0, 2, 3, 4, 5, 6) for all joints.

**Table 1:** The Medical Research Council (MRC) [48] scale grade with modifications based on the Kumamoto scale [32] and the Neuropathy Impairment Score (NIS) [25]

Kumamoto scale score*	Description
0	Normal power (NIS-LL 0; MRC: 5)
2	50% weak (NIS-LL 2; MRC: 4)
3	Active movement against gravity (NIS-LL: 3.25; MRC: 3)
4	Active movement with gravity eliminated (NIS-LL: 3.50, MRC: 2)
5	Visible contraction but no limb movement (NIS-LL: 3.75, MRC: 1)
6	No visible contraction (NIS-LL: 4, MRC: 0)

^*^Modified MRC

#### Scoring of visceral organ impairment

Proteinuria is tested for with urine dipsticks and can be supplemented with a urine protein/creatinine ratio. Nephrotic syndrome is defined as proteinuria (>3.5 g/24 h), hypoalbuminemia, and oedema. We suggest changing “Renal failure: need for dialysis” to “On dialysis” since the need for both haemodialysis and peritoneal dialysis suggest end-stage renal failure. Unfortunately, we found it difficult to revise the scoring for cardiac impairment without interfering too much with the original score. Thus, the scoring was left unchanged ranging from 0 to 12 points with four subscores (0, 4, 8, and 12) for both heart and kidney involvement.

### The revised Kumamoto scale

**Fig. 5:**
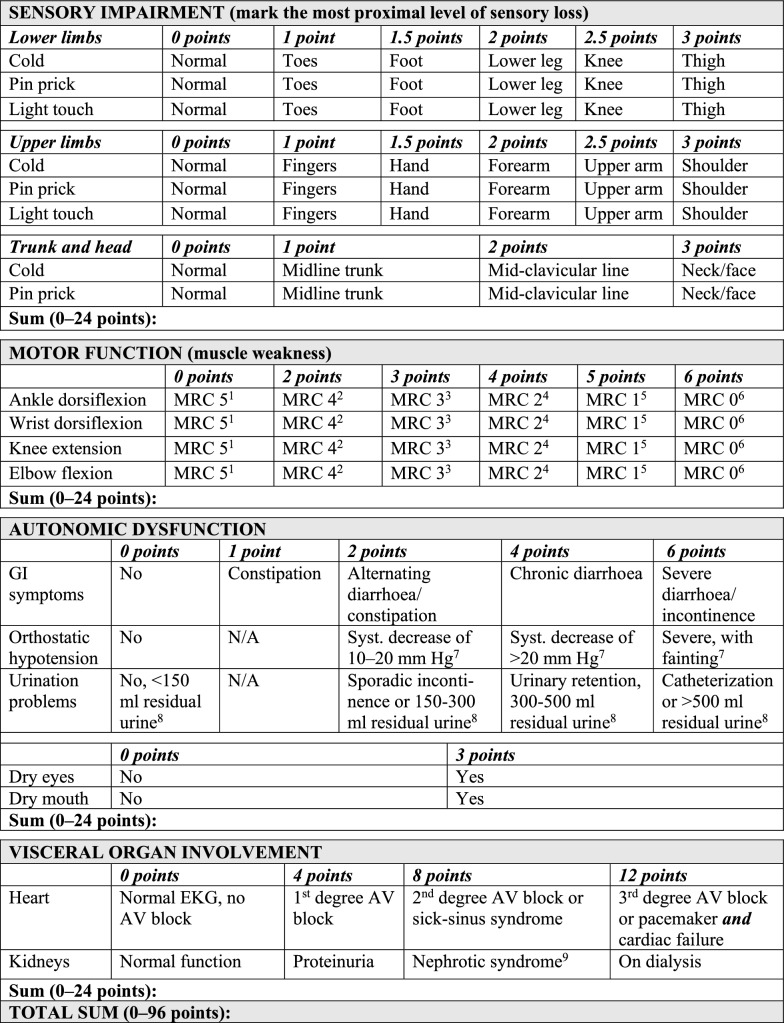
The revised Kumamoto scale with subscores for the different domains and the total score left unchanged. ^1^Normal power, ^2^Movement against resistance, 50% weak, ^3^Movement against gravity, ^4^Movement with gravity eliminated, ^5^Visible contraction but no movement, ^6^No visible contraction, ^7^Measured after 1 and 3 min in a standing position. ^8^Verified by a bladder scan post voiding. ^9^Proteinuria (>3.5 g/24 h), hypoalbuminemia and oedema. MRC: Medical Research Council; N/A: not applicable

Based on the discussion above, the final version of the revised Kumamoto scale is presented in Fig. 5. The subtotal (0–24 points) and total scores (0–96 points) have been left unchanged, although the scoring for some specific items have been changed.

### Comparison of the original and the revised Kumamoto scale

In Table 2, the scoring for a quite typical ATTRv amyloidosis patient with moderate sensory polyneuropathy (loss of cold sensation in feet, lower legs and fingers, and loss of pinprick sensation in the feet), normal motor function, a systolic blood pressure decrease of 5 mm Hg after standing for 3 min, chronic constipation (two hard stools per week), dry mouth, proteinuria on urine dipsticks, and a first-degree AV block on ECG is displayed for both the original and the revised Kumamoto scale.

**Table 2:** Comparison of the scoring for a patient with sensory neuropathy in fingers (loss of cold sensation), feet (loss of cold and pin prick sensation) and lower legs (loss of cold sensation), normal motor function, a systolic blood pressure decrease of 5 mm Hg after standing in 3 min, chronic constipation (two hard stools per week), dry mouth, proteinuria on urine dipsticks, and a first-degree AV block on ECG between the original and the revised version of the Kumamoto scale

	Original scale score	Revised scale score
*Lower limbs*		
Cold sensation	2	2
Pinprick sensation	1	1.5
Light touch sensation	0	0
*Upper limbs*		
Cold sensation	1	1
Pinprick sensation	0	0
Light touch sensation	0	0
*Trunk and head*		
Cold sensation	0	0
Pinprick sensation	0	0
Sensory subscore (0–24 points)	4	4.5
*Motor function*		
Ankle dorsiflexion	0	0
Wrist dorsiflexion	0	0
Knee extension	0	0
Elbow flexion	0	0
Motor subscore (0–24 points)	0	0
*Autonomic dysfunction*		
GI symptoms	0	1
Orthostasis	2	0
Urination	0	0
Dry eyes	0	0
Dry mouth	3	3
Autonomic subscore (0–24 points)	5	4
*Visceral organ involvement*		
Heart	4	4
Kidneys	4	4
Visceral organ subscore (0–24 points)	8	8
**Total score (0–96 points)**	**17**	**16.5**

## Discussion

Given the multisystemic and progressive nature of ATTRv amyloidosis, there is a strong need for standardised and easy-to-use tools for follow-up of ATTRv patients. This is even more important today when several disease-modifying therapies are available. Although attempts have been made, there is still no true consensus on which tool(s) to use for monitoring of ATTRv amyloidosis [26–31]. This is probably due to the disease complexity, with a wide range of genotypes and different phenotypes, as well as to the differences in healthcare resources between clinics and countries.

There is probably not any stand-alone clinical instrument that can be used for easy and accurate monitoring of the progressive sensorimotor and autonomic polyneuropathy and visceral organ involvement seen in ATTRv amyloidosis. A combination of tools may be needed for patient follow-up, and the different phenotypes might require different tools. As the Kumamoto scale was developed for systematic evaluation of ATTRv patients, it covers most aspects (autonomic and sensorimotor polyneuropathy and visceral organ involvement) of the disease [32, 34]. The scale has been used in both clinical trials and clinical practice during the last 20 years [13, 33] and is nationally recommended for patient follow-up in Sweden [35] and Denmark—the two Nordic countries with clustering areas of ATTRv amyloidosis. Our clinical experience is that the scale is practical to use and covers the most important aspects of the disease, although it comes with some limitations and has mainly been evaluated in patients with ATTRV30M amyloidosis. The scoring system allows for easy evaluation of the disease manifestations over time if the potential sources of error are kept in mind. Overall, the Kumamoto scale is easier to use in clinical practice than the NIS and mNIS scores.

After deciding to revise the Kumamoto scale at the Nordic Amyloidosis Day in 2023, we identified its main limitations and tried to revise the scale with as little impact on the content and scoring as possible. Our focus has been to homogenise the different parts of the scale to make it more granular and to standardise the measurements and add any important missing symptoms. In our final revision, the levels of sensory loss in the limbs have been refined and the subscores have been changed to a 5-point scale to allow for a more fine-tuned sensory evaluation. Moreover, the evaluation of motor function has been more closely defined, and a standardised assessment method (the MRC scale) has been implemented. Furthermore, constipation has been added as a symptom of autonomic dysfunction, and the cut-off for orthostatic hypotension has been increased from 0 to 10 mm Hg. The subtotal scores and the total score have been left unchanged to allow for retrospective comparisons with the original Kumamoto scale.

Altogether, we believe that our revision has significantly improved the Kumamoto scale, and that this publication will help clarify its strengths and limitations. Still, the scale is not fully comprehensive since some disease manifestations are not included. For example, cardiac symptoms and signs of heart failure, upper GI symptoms, and genitourinary symptoms are missing, and the assessment of visceral organ involvement is rather blunt; however, this should always be weighed against the user-friendliness of the scale. The current revised version of the Kumamoto scale is not more complex than the original scale, which was part of our goal with the revision. One may argue that the scoring is a bit skewed within the subdomains, although we have tried to homogenise this as much as possible. The fact that the maximum subtotal score is the same (24 points) for each subdomain is a strength, given the large phenotypic variation of the disease.

In our experience, the Kumamoto scale is a good and validated tool for clinical follow-up of ATTRv amyloidosis that should be combined with a detailed medical history, a general physical examination, biomarkers (albumin, creatinine, troponin T or I and nt-proBNP), nutritional status (modified body mass index, mBMI), and some functional status scales like the Karnofsky performance scale, the New York Heart Association (NYHA) scale, and the Polyneuropathy Disability (PND) scale to compensate for its limitations. Clinical follow-up should be performed annually or biannually depending on the patients’ status and genotype, as well as on local healthcare resources. According to Swedish guidelines, significant disease progression is defined as subjective deterioration together with >4 points increase on the Kumamoto score, unintentional weight loss of at least 5% of the body weight, and/or onset of new symptoms or signs of further organ involvement (the eyes excluded) [35].

Additionally, more specific examinations like nerve conduction studies, heart rate variability tests, gastric emptying scintigraphy, Holter ECG, echocardiography, and cardiac MRI and/or cardiac scintigraphy with bone tracers should also be performed according to current national or local guidelines. In our opinion, Holter ECG should be performed annually to capture any significant arrhythmias, whereas echocardiography can be performed every other year to evaluate the progression of amyloid cardiomyopathy. Nerve conduction studies should be performed at least at diagnosis and on suspicion of a worsening of the peripheral polyneuropathy.

Although we have revised and tried to improve the Kumamoto scale, our approach comes with some limitations. Our expert group consists of a smaller group of specialists from the Nordic countries, which is a potential source of bias. Of course, a broader international consensus among a larger number of specialists would be preferable. Also, the scale has not been directly compared to other clinical tools used for ATTRv amyloidosis, however, the original Kumamoto scale showed similar outcomes as NIS and NIS + 7 in the pivotal diflunisal trial [13]. Further, we have not yet performed any validation studies but plan to assess the intra- and interrater variability in a smaller number of patients as a next step.

In conclusion, we believe that the revised Kumamoto scale is a reliable and easy-to-use clinical tool for monitoring ATTRv amyloidosis that is suitable for use in clinical trials. If combined with selected disease biomarkers, nutritional status, and functional status scales, it offers a comprehensive clinical assessment of ATTRv patients. However, a validation of the revised scale would further strengthen our results.

## Data Availability

No new data available.

## Abbreviations

ATTRv: Hereditary transthyretin amyloidosis
AV: Atrioventricular
ECG: Electrocardiogram
GI: Gastrointestinal
MRC: Medical Research Council scale
MRI: Magnetic resonance imaging
NIS: Neuropathy Impairment Score
NIS-LL: NIS lower limbs
TTR: Transthyretin
NYHA: New York Heart Association scale
PND: Polyneuropathy Disability scale
